# Advances in the Protective Mechanism of NO, H_2_S, and H_2_ in Myocardial Ischemic Injury

**DOI:** 10.3389/fcvm.2020.588206

**Published:** 2020-10-30

**Authors:** Wei-lu Wang, Tian-yu Ge, Xu Chen, Yicheng Mao, Yi-zhun Zhu

**Affiliations:** ^1^Guilin Medical College, Guilin, China; ^2^Shanghai Key Laboratory of Bioactive Small Molecules, Department of Pharmacology, School of Pharmacy, Fudan University, Shanghai, China; ^3^State Key Laboratory of Quality Research in Chinese Medicine and School of Pharmacy, Macau University of Science and Technology, Macau, China

**Keywords:** myocardial ischemia, NO, H_2_S, H_2_, gas co-dornor, protecting mechanisms

## Abstract

Myocardial ischemic injury is among the top 10 leading causes of death from cardiovascular diseases worldwide. Myocardial ischemia is caused mainly by coronary artery occlusion or obstruction. It usually occurs when the heart is insufficiently perfused, oxygen supply to the myocardium is reduced, and energy metabolism in the myocardium is abnormal. Pathologically, myocardial ischemic injury generates a large number of inflammatory cells, thus inducing a state of oxidative stress. This sharp reduction in the number of normal cells as a result of apoptosis leads to organ and tissue damage, which can be life-threatening. Therefore, effective methods for the treatment of myocardial ischemic injury and clarification of the underlying mechanisms are urgently required. Gaseous signaling molecules, such as NO, H_2_S, H_2_, and combined gas donors, have gradually become a focus of research. Gaseous signaling molecules have shown anti-apoptotic, anti-oxidative and anti-inflammatory effects as potential therapeutic agents for myocardial ischemic injury in a large number of studies. In this review, we summarize and discuss the mechanism underlying the protective effect of gaseous signaling molecules on myocardial ischemic injury.

## Introduction

Ischemic injury is caused mainly by anerobic cell death and reperfusion (1). Endothelial dysfunction, microvascular collapse, and blood flow defects are preconditions for phenotypic expression of ischemic injury (2), which is mediated by a variety of cytokines, chemokines, and adhesion molecules, as well as extracellular matrix compounds (3). Ischemic injury causes damage to a variety of organs and tissues, such as the brain (4, 5), liver (6), intestines (7), limbs (8), and heart (9). Myocardial ischemic injury is one of the most common and serious diseases that endangers human health. It is usually caused by coronary artery stenosis or occlusion caused by coronary atherosclerosis. Acute and temporary myocardial ischemia and hypoxia can cause angina pectoris. Persistent and severe myocardial ischemic injury can cause myocardial necrosis or myocardial infarction (MI) and even heart failure (HF).

In this review, we introduce gaseous signaling molecules and summarize the mechanism of their protective effects against myocardial ischemic injury. The gaseous signaling molecules include NO, H_2_S, H_2_, the gas joint donor, such as ZYZ-803, and other gas donor molecules. The aim of this review is to provide a more comprehensive understanding of gaseous signaling molecules and to promote further research to clarify their potential clinical application for the prevention and treatment of myocardial ischemic injury.

## Treatment of Myocardial Ischemic Injury

The current, treatments for myocardial ischemic injury mainly include surgical, and drug approaches. Surgical treatments include percutaneous coronary intervention (PCI) (10) and coronary artery bypass graft (CABG) (11). PCI is a procedure used to improve myocardial perfusion by cardiac catheterization to open a narrow or even occluded coronary artery lumen (12). CABG refers to the establishment of a vascular pathway between the root of the ascending aorta and the distal obstruction of the diseased coronary artery using transplanted blood vessels, to achieve blood flow recanalization by bypassing the lesion site in the coronary artery (13). Drug therapies include conventional Western drugs and conventional Chinese medicines. The main Western drugs such as the anti-platelet drug aspirin, and nitroglycerin, used to treat myocardial ischemic injury are listed in Table 1. Traditional Chinese medicines, include heart-protecting musk pill, which has a role in preventing ventricular remodeling in patients with acute MI (22) and in protecting against arteries atherosclerosis (23), and qishen yiqi drop pill (QSDP), which has a role in improving cardiac function after myocardial ischemic (24). At present, however, traditional Chinese drugs are generally not recommended as the main treatment for myocardial ischemic, although they can be used as an adjunct to Western medicine therapy. Although conventional drugs have been used, the plethora of side effects and contraindications, as well as the limited availability of raw materials, have led researchers to focus on new drug candidates.

**Table 1:** The main traditional Western medicine used for myocardial ischemic injury.

**Compound**	**Mechanisms**	**Advantages**	**Disadvantages**	**References**
Aspirin	Inhibition of platelet function	Extensive use: treatment of fever, pain and rheumatoid arthritis and etc.	More adverse reactions	(14)
Clopidogrelg	Inhibition of platelet function	Extensive use: treatment of fever, pain and rheumatoid arthritis and etc.	Causes neutropenia or thrombocytopenia	(15)
Heparin	Anticoagulation	Broad indications	The anticoagulant effect varies greatly among individuals; Prone to causing embolism re-occlusion, osteoporosis and thrombocytopenia	(16)
Nitroglycerin	Reduction of cardiac load	Treatment or prevention of angina pectoris; Used as a asodilator for the treatment of congestive heart failure	Many contraindications	(17)
Metoprolo	Reduction in myocardial oxygen consumption, reduce cardiac load	Broad indications	Many contraindications	(18)
Captopril	Prevention of myocardial remodeling	Anti-hypertension	Partially blocks the generation of angiotensin II; A dry cough	(19)
Losartan	Prevention of myocardial remodeling	Antihypertensive; Well-tolerated	Many contraindications	(20)
Simvastatin	Increased lipoprotein lipase; Reduced cholesterol synthesis	Lower cholesterol, low density lipoprotein cholesterol, and very low density lipoprotein cholesterol	Muscle toxicity; Elevated liver enzymes; Causes adverse, the symptoms in the nervous and gastrointestinal systems	(21)

## Source of Gaseous Signaling Molecules

Current studies have shown that gas signaling molecules mediate certain inhibitory effects on oxidative stress (25), apoptosis (26), inflammation (27), and autophagy (28), and have protective effects on many organs, including the heart (29). Compared with conventional drugs, gas signaling molecules have smaller molecular weights that facilitate entry of the biofilm. Their effects are independent of the corresponding membrane receptors and cytological effects, and may or may not depend on mediation by a second messenger. Gas signaling molecules are also derived from a wide variety of sources in nature, such as garlic and onion, and they play an important role in humans. Pan et al. (30) reported that the sulfide S-ally-L-cysteine (SAC) contained in garlic can be used as an H_2_S donor drug for cardiac protection. Specifically, garlic has been shown to mediate cardiac protective effects such as reduction of the blood lipids and blood pressure, and is a source of antioxidants (which scavenge free radicals and inhibit lipid peroxidation) (31). Furthermore, it has been reported that the antioxidant effect of garlic may be associated with “nucleophilic tone,” which is defined as “the capacity to remove electrophiles through enzyme catalyzed, the dynamic flow of reducing equivalents from NADPH, glutathione (GSH) and reduced thioredoxin (32).” Zhao et al. (33) showed that allicin in garlic alleviated myocardial ischemic injury by promoting autophagy. In 1990, Makheja et al. (34) identified adenosine, allicin and paraffin-based polysulfide as three main anti-platelet components of onion. Among them, adenosine acts as a trigger and regulator of cardioprotection (35). Moreover, Park et al. (36) found that a methanol extract of onion alleviated myocardial ischemic injury by reducing the ROS content of hypoxic cardiomyocytes. Thus, gaseous signaling molecules like NO, H_2_S, and H_2_ have strong potential in the protection of the heart against myocardial ischemic injury. NO, H_2_S, and H_2_ are gases at room temperature and exhibit unique properties and functions in nature and living organisms including organs such as the heart.

## Mechanism of Myocardial Ischemic Injury

### Free-Radical Action

#### The Mechanism Underlying the Increase in Oxygen Free-Radicals in Myocardial Ischemia

**Figure 1:**
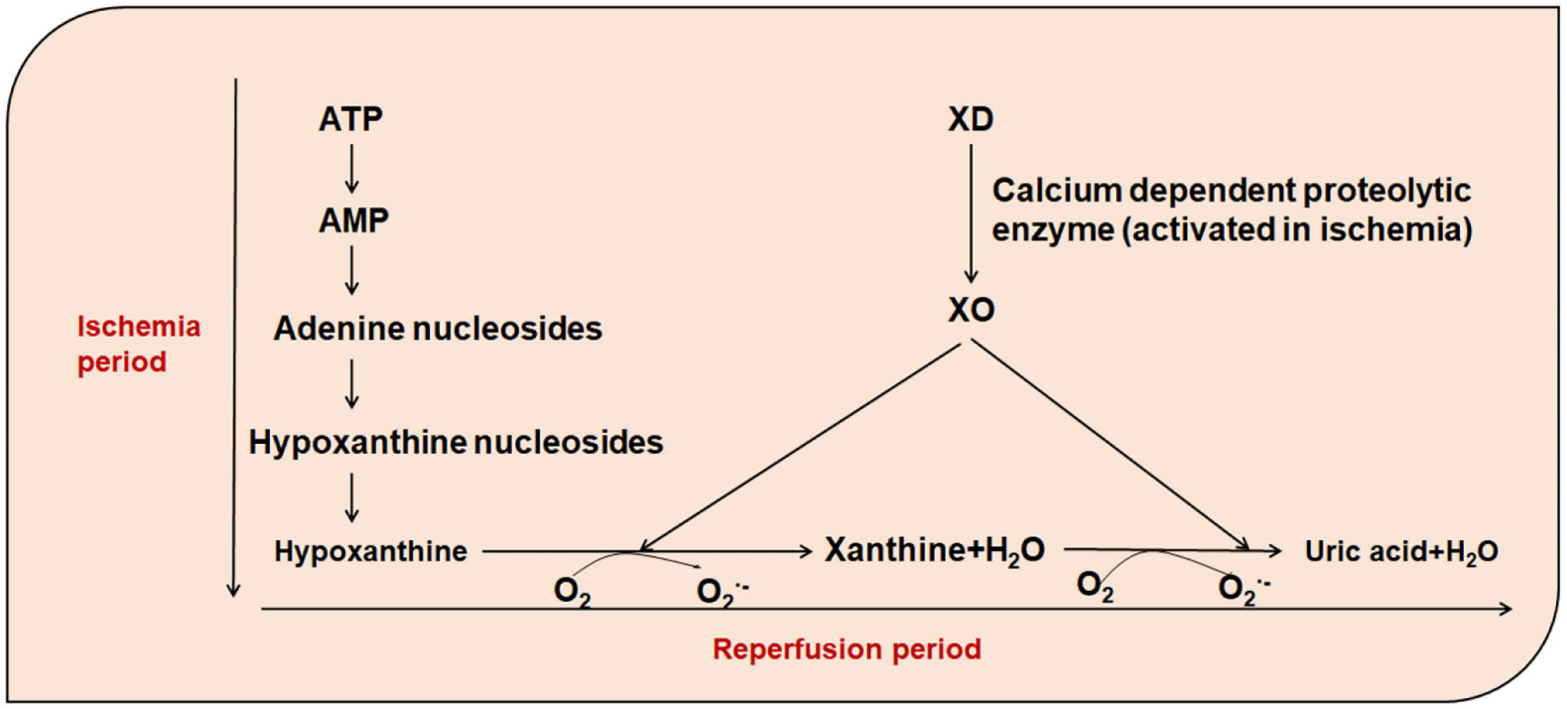
The role of xanthine oxidase in the presence of increased free-radicals. ATP, Adenosine triphosphate; AMP, Adenosine monophosphate; XO, Xanthine oxidase; XD, xanthine dehydrogenase; O_2_, oxygen; ${\text{O}}_{2}^{.-}$, Superoxide Anion.

Xanthine oxidase (XO) and xanthine dehydrogenase (XD) are present in cardiomyocytes. XO is present in only 10% of normal cardiomyocytes, while XD is present in 90%. During coronary atherosclerosis or coronary artery embolization, mitochondrial permeability changes in the human cells lead to matrix swelling, rupture of the outer membrane, release of apoptotic signaling molecules, and irreversible damage to mitochondria (37). Under conditions of myocardial ischemia, on the one hand, dysfunction of the Ca^2+^ pump and changes in the oxidation state of thiols due to the decrease in ATP, XD is transformed into large amounts of XO in a reaction catalyzed by a Ca^2+^-dependent proteolytic enzyme. On the other hand, due to the decrease in oxygen partial pressure, ATP is degraded to ADP, AMP, and hypoxanthine, which accumulates in ischemic tissues. During treatment of myocardial ischemia, the process is often accompanied by reperfusion. During reperfusion, a large amount of molecular oxygen enters into the ischemic tissues along with the blood. XO once again catalyzes the conversion of hypoxanthine to xanthine and further catalyzes the conversion of xanthine to uric acid, thus producing large amounts of ${\text{O}}_{2}^{\text{.}-}$ and H_2_${\text{O}}_{2}^{.}$ Furthermore, the reaction of ^.^OH formed with the participation of metal ions is more intense with the diffusion control of most molecules (38) (Figure 1). In addition, hypoxia leads to decreased oxygen partial pressure and ATP production in cells, increased entry of calcium ions into the mitochondria, dysfunction of mitochondrial oxidative phosphorylation, electron transport chain damage, increased entry of oxygen free-radicals into cells, and reduced Mn-SOD, leading to reduced free-radical scavenging capacity, and therefore, increasing the local level of free-radicals. In addition, free radicals are produced by increased NADPH oxidase and peroxidase activity and catechol-amine autoxidation.

#### The Mechanism by Which Free-Radicals Cause Myocardial Ischemic Injury

Dysfunction in free-radical removal systems, such as SOD enzymes, catalase, and ascorbic acid, results in excessive free-radical production that causes damage to biological macromolecules, such as nucleic acids, proteins and lipids, affecting their normal physiological functions. Cellular lipids, proteins and DNA also react directly with free-radicals, causing damage to the cell structure and dysfunction, accompanied by activation of the NF-κB signaling pathway (39).

### Intracellular Calcium Ion Overload

**Figure 2:**
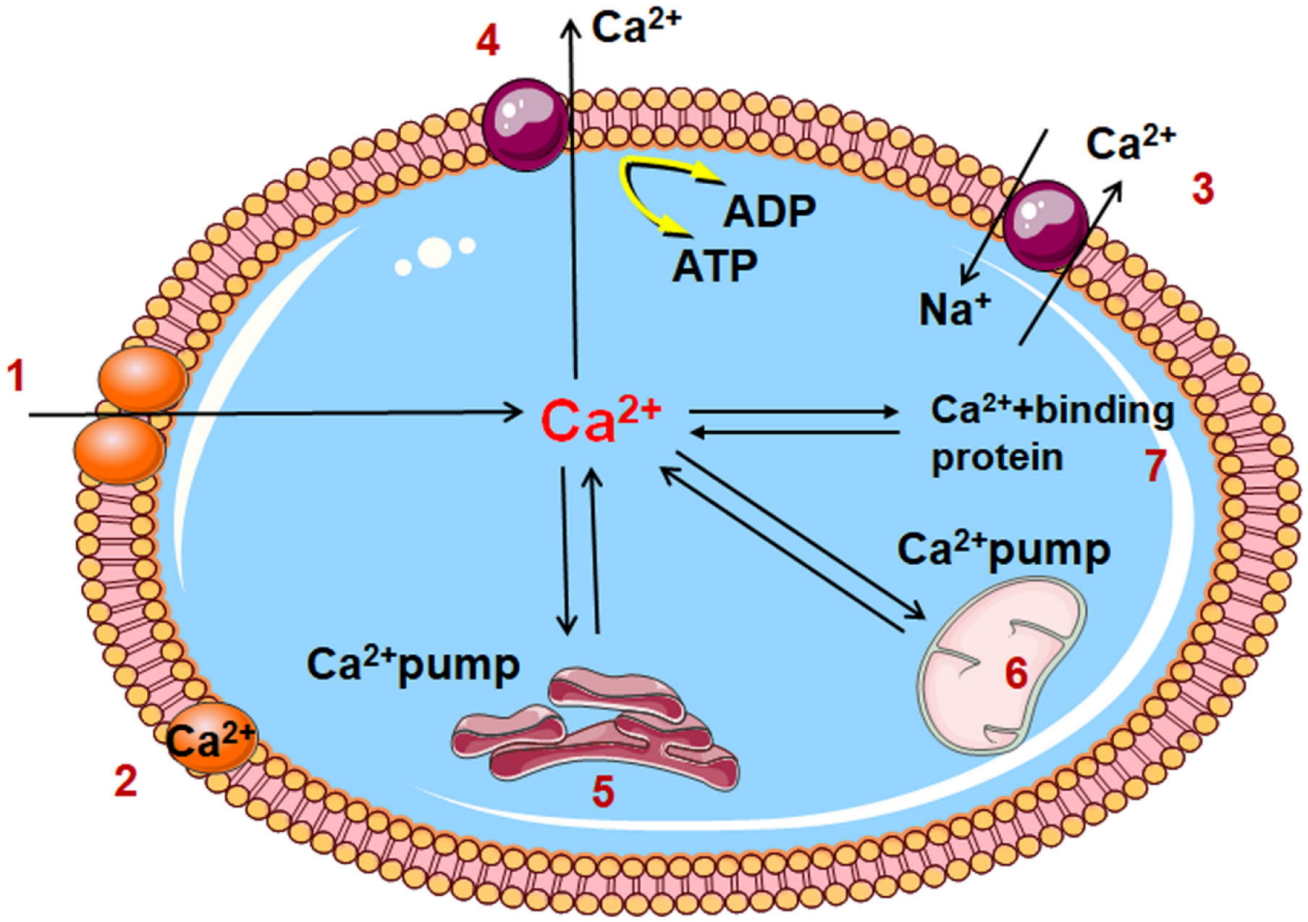
Diagram of cell calcium transport pattern 1. Voltage dependent calcium channel; 2. Cell membrane binding calcium; 3. Na^+^/ Ca^2+^ exchange; 4. Calcium channel of cell membrane 5. Sarcoplasmic reticulum; 6. Mitochondria; 7. Cytoplasmic binding calcium; A TP, Adenosine triphosphate; ADP, Adenosine diphosphate.

Under normal conditions, the extracellular calcium ion concentration is 10,000 times higher than that inside the cell; a schematic diagram of the process of the transport of calcium ion transport is shown in Figure 2. Under conditions of myocardial ischemia, changes in intracellular and extracellular Ca^2+^ regulation lead to Ca^2+^ overload in the cytoplasm and mitochondrial matrix. The myocardial structure is damaged, function is reduced or electrophysiological disorders are caused by excessive contraction of energy-dependent muscle fibers. In addition, cell proteolysis is mediated by calpain, mitochondrial permeability transition pores are opened, inducing cell apoptosis and closure of gap junction channels, rendering cell activity out of synchronization with other pathways. Abnormal Na^+^-Ca^2+^ exchange (40), protein kinase C activation (41), and increased intracellular calcium ion levels during biofilm injury can lead to calcium ion overload in cardiac myocytes. Furthermore, the aggregation of intracellular calcium ions results in phospholipase activation and degradation, further increasing the permeability of cell membrane to calcium ions, and promoting membrane damage. In addition, during myocardial ischemia-reperfusion, intracellular calcium ion overload may cause excessive contraction of myocardial fibers, leading to arrhythmias and exacerbating the symptoms of myocardial injury.

### Endothelial Cells Appear to Have Intercellular Gaps Through Which White Blood Cells Migrate

Inflammation, which is the response of the body to pathogenic factors, comprises injury and anti-injury processes. Inflammatory mediators, such as histamine and bradykinin, adhesion of leukocytes, release of proteolytic enzymes and active oxygen metabolites, can cause endothelial cell mediating contraction/dilation of underlying smooth muscle cells, and movement between cells, resulting in increased osmotic pressures of cell crystals and colloids. At this point, the white blood cells migrate through the intercellular gaps. In experimental myocardial ischemic injury, pro-inflammatory cytokines, including TNF-α, IL-6, IL-1, participate in post-ischemic responses (42), although some inflammatory cytokines, such as COX1, mPGEs1, also protect against post-ischemic response (43).

## Mechanism Underlying the Role of no in Myocardial Ischemic Injury

### NO and the Myocardium

NO is a gas at room temperature, although it acts as a first messenger due to its fat-solubility. NO is also known as endothelium-derived vasodilator factor (EDRF). As a gaseous signaling molecule that is known for its vasodilatation effects (44). NO is a free-radical, which is relatively stable compared to most species, and exhibits a select spectrum of rapid and life-limiting reactions in biological systems. *In vivo*, NO synthesis requires L-arginine, oxygen, and NOS, in addition to the cofactors tetrahydrofolate (BH_4_), NADPH, flavin adenine dinucleotide (FAD), flavin mononucleotide (FMN), heme and calmodulin (45). Thus, NO synthesis from L-arginine and dioxygen is a multistep process catalyzed by the mammalian NOS isoenzymes in a unique active site constructed around heme and BH_4_ cofactors. NO is otherwise unable to dissociate from ferrous heme. The shift in the potential of the heme on NO binding from −300 to 0 mV in the presence of substrate indicates that the dissociation constant for NO binding to ferrous nNOSoxy is 0.17 nM. A similar interaction between substrate and heme-bound dioxygen increases the kinetic stability of the oxyferrous complex, slowing the rate of decay via electron transfer from BH_4_ or via superoxide release (46). There are three subtypes of NOS: endothelial (e-NOS), neuronal (n-NOS), and inducible NOS (i-NOS) (47). e-NOS is highly expressed in coronary vessels and endocardial endothelial cells (48). e-NOS/NO signaling can reduce the area of myocardial ischemia to alleviate adverse cardiac remodeling (49) and inhibit ROS and angiotensin II (ANG II)-induced endothelial cell apoptosis (50). NO also serves as an effective regulator of blood pressure and blood flow (51). The protective effect of NO on endothelial cells has also been confirmed in mice with e-NOS damage. The changes of NO are closely related to hypertension (52) and coronary heart disease (53). However, in the presence of e-NOS/NO, DNA synthesis in smooth muscle cells is inhibited (54, 55).Similar to e-NOS, n-NOS is also expressed in the cardiovascular system, primarily in the sarcoplasmic reticulum (56), although a small fraction is expressed in the mitochondria (57), Golgi bodies (58), and myofilms (57). n-NOS plays a key role in protecting the myocardium from oxidative stress, systolic/diastolic dysfunction, poor structural remodeling, and arrhythmias in failing hearts (59, 60). Furthermore, n-NOS/NO regulates cardiac electrophysiology and intracellular Ca^2+^ protein homeostasis, targeting myosin through S-nitrosylation and phosphorylation, dynamic regulation of mitochondrial activity and biological activity (61). i-NOS is expressed in cardiomyocytes in response to specific cytokines. The i-NOS/NO signaling pathway can reduce the occurrence of obvious HF, but excessive expression often causes cardiomyopathy, arrhythmias and even sudden cardiac death (62). In addition, NO metabolites include nitrite, which is an important repository of NO in blood and tissue, and nitroso mercaptan (63, 64). NO and nitroso mercaptan levels are reduced in ischemia or hypoxia (65). Circulating levels of these metabolites directly regulate their tissue storage (66), and increasing levels of nitrite and nitrosothiol in the heart is an effective cardiac protection strategy (67, 68). Currently, common exogenous NO donors include NaNO_2_ and furoxan, which releases NO in the presence of mercaptan.

### Oxidative Stress and NO

The updated definition of “oxidative stress” is “an imbalance between oxidants and antioxidants in favor of the oxidants, leading to a disruption of redox signaling and control and/or molecular damage (69).” In general, NO has a protective effect on the damage caused by myocardial ischemia when appropriate amounts of are in equilibrium with antioxidants and oxygen free radicals (70). Tan et al. (71) demonstrated that NO is an effective antioxidant experimentally by evaluating the cardiac protective function of baicalein in rats with acute myocardial infarction (AMI). Furthermore, the possible molecular mechanism was explored by administration of baicalein and/or e-NOS inhibitor L-NAME before inducing AMI. Analysis of the corresponding indicators, such as creatine kinase, creatine kinase MB isoenzyme, lactate dehydrogenase and cardiac troponin T revealed that baicalin activated the e-NOS signaling pathway and inhibited oxidative stress in rats with AMI through e-NOS signal transduction. Xiao et al. (72) reported the same protective effect of NO in another study using luteolin as the experimental drug, in an experimental model of myocardial ischemic injury induced by diabetes. It was found that after activation by luteolin, e-NOS inhibited the binding of Nrf2 to Keap1, Nrf2 was then transferred to the nucleus, where it combined with antioxidant genes, such as ARE, to produce an antioxidant effect (72).

However, excessive NO can have harmful effects on the heart muscle. For example, Tao et al. (73) simulated myocardial ischemia-reperfusion *in vivo* by ligating the coronary arteries of mice for 30 min followed by reperfusion for 3 or 24 h. Comparison of the myocardial infarct size in mice, production of peroxynitrite, NO and superoxide as well as i-NOS and gp91^phox^ protein expression, it was found that the adiponectin globular domain structure reduced the myocardial ischemia/reperfusion induced i-NOS/gp91^phox^ protein expression, reduced the generation of NO/peroxide, blocked oxygen nitrite formation, and reversed adiponectin^−/−^ mice to expand the infarction effect. Thus, it is clear that the balance of NO content is a very important factor for regulating ischemic injury.

### Anti-apoptotic Effects and NO

Accumulating evidence shows that the NO system plays a key role in the regulation of myocardial cell apoptosis (74, 75). For example, Wang et al. (76) found that ginsenoside Rg3 (GSRg3) mediated myocardial protection and inhibited MI/R-induced apoptosis by up-regulating the Akt/e-NOS/NO signaling pathway. Liu et al. (77) found that hydromorphine administered post-treatment protected the isolated rat heart from reperfusion injury by activating P13K/Akt/e-NOS signal transduction. Moreover, downregulation of MIR-134 was also shown to activate the P13K/Akt/e-NOS signaling pathway to protect muscle I/R injury (78). Increased intracellular calcium production is the main mediator of myocardial cell apoptosis induced by ischemia (79). Therefore, the restoration of intracellular calcium inflow can effectively prevent ischemic myocardial cell apoptosis. Li et al. (79) showed that KMUP-1 [7-[2-[4-(2-chlorophenyl) piperazinyl] ethyl]−1] activated e-NOS expression and restored the intracellular calcium flow by upregulating the NO/cGMP/MAPK signaling pathway, which inhibited the apoptosis induced by myocardial ischemia. Similarly, phosphorylation and expression of e-NOS were increased.

### The Anti-inflammatory Effects of NO

Inhibition of myocardial inflammation and pathological remodeling after MI injury is very important for the treatment of ischemic cardiomyopathy. The inflammatory factor COX, which is a rate-limiting enzyme of prostaglandin (PG), catalyzes the conversion of arachidonic acid to PGH_2_ (80). COX exists as COX1 in large amounts of normal cells and COX2, which is closely related to NO associated signaling pathways, is induced by stress (80). Shinmura et al. (81) showed that NO protects the ischemic myocardium by stimulating COX2 to produce cell-protective prostaglandins, such as PGE2 and PGI. In the later pretreatment, it was found that inhibition of i-NOS eliminated prostaglandin synthesis, while inhibition of COX2 had no effect on i-NOS activity (81), but resulted in loss of the protective effect, indicating that the activity of COX2 activity was driven by i-NOS. Pang et al. (82) found that COX2 inhibited AKT formation and promoted the production of NO in i-NOS, thereby preventing H9C2 cells harm due to hypoxia/reoxygenation. In addition to i-NOS, e-NOS also plays an important role in the anti-inflammatory pathway. For example, O-nitrophenyl ethyl caffeate (CAPE-oNO2) inhibits inflammation following myocardial ischemia-reperfusion injury via the e-NOS/NF-kB pathway (83).

In summary, a large amount of evidence (82–85) indicates that NO exerts an anti-inflammatory effect on MI by regulating the activity of inflammatory cytokines or being regulated by inflammatory cytokines.

## Mechanism of H_2_S Protection Against Myocardial Ischemic Injury

### Induction of H_2_S

Hydrogen sulfide (H_2_S) was the second gaseous signaling molecule discovered after NO. As with NO, the role of H_2_S in organs or tissues such as the kidney (86, 87), brain (88, 89), and heart (90, 91) has been documented and has been shown to play an important role in the regulation of cardiovascular activities. H_2_S is a colorless gas with the odor of rotten eggs (92). *In vivo*, the main enzymes responsible for H_2_S synthesis are cystathionine γ-lyase (CSE), cystathionine β-synthase (CBS) and 3-mercaptopyruvate sulfurtransferase (3-MST) (93). Yang et al. (62) confirmed that CSE is mainly expressed in the cardiovascular system by preparing CSE gene-deficient mice and measuring the H_2_S content. H_2_S levels in the aorta and arterioles were decreased by ~50 and 80%, respectively, in CSE-deficient mice compared with those in the wild-type mice and the serum H_2_S levels were also decreased by 50%, indicating that CSE is the main source of H_2_S in the cardiovascular system. Numerous studies (94–99) have shown that H_2_S preconditioning can significantly antagonize MI injury, reduce the MI area, reduce troponin I levels and the lever of oxidative stress, apoptosis and inflammation. Calvert et al. (99) showed that H_2_S increased the nuclear translocation of Nrf2 and upregulated the phosphorylation of PKCε and STAT3 in the early stage of pre-treatment. In the later stage of H_2_S pre-treatment stage, the expression of HO-1, thioredoxin 1 and heat shock protein 90 (HSP90) increased, and the activity of pro-apoptotic factors decreased (99). However, H_2_S does not usually play a direct antioxidant role. H_2_S is first dissociated as HS^−^, then HS^−^ and then S^2−^ in solution. The percentages of HS^−^ and H_2_S in solution are 81 and 19%, respectively, while the concentration of S^2−^ is almost negligible, indicating that most of the influence of H_2_S is mediated by thiols (100).

**Figure 3:**
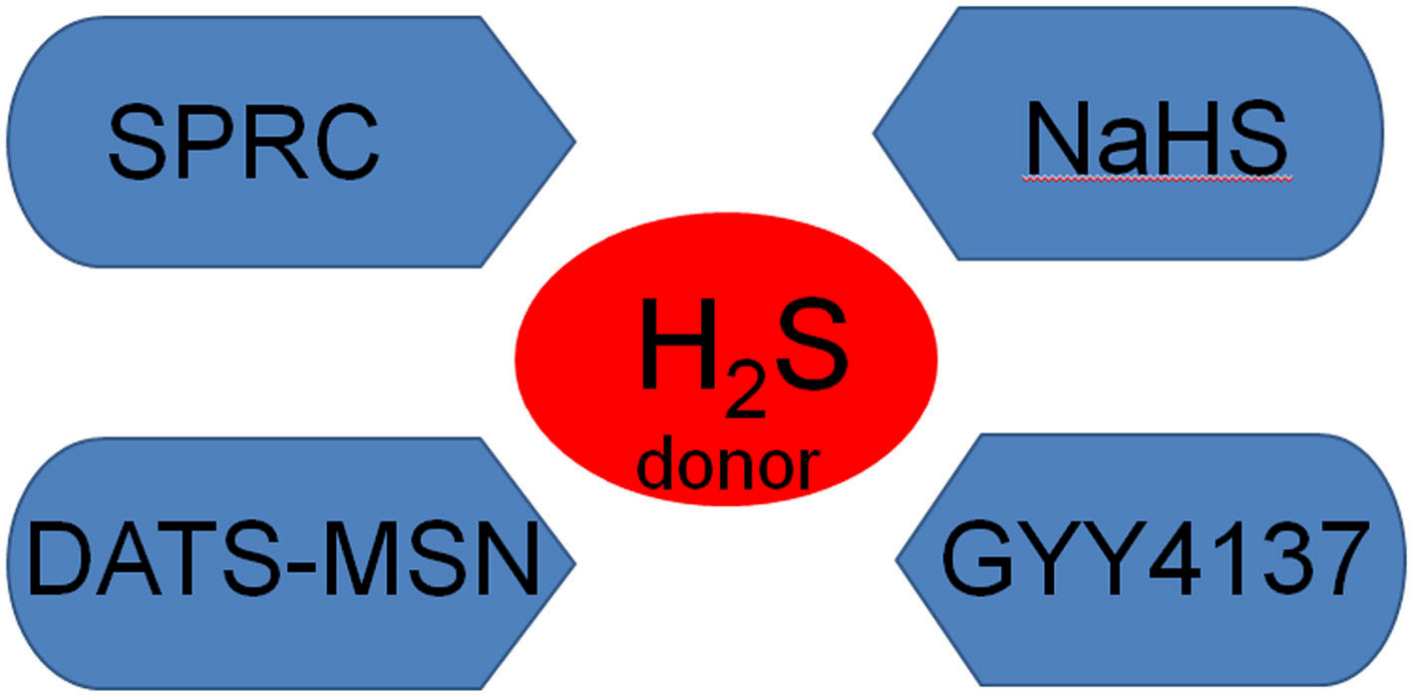
The main H_2_S donors NaHS, sodium hydrosulfide; GYY4137, morpholine-4-methoxyphenylmorpholine-morpholine-phosphodisulfate; SPRC, s-propargyl-cystine.

*In vitro*, commonly used H_2_S donors include NaHS, morpholine-4-methoxyphenylmorpholine-morpholine-phosphodisulfate (GYY4137), DATS-MSN and s-propargyl-cystine (SPRC), all of which have protective effects against MI. NaHS is the most commonly used H_2_S donor *in vitro*. For example, Li et al. (94) used NaHS as the H_2_S donor to demonstrate that H_2_S pre-treatment reduced ER/SR stress in the hypoxia/reoxygenation model in H9C2 rat cardiomyocytes and inhibited cardiomyocyte apoptosis. Similarly, Wang et al. (101) used NaHS as a donor to study the protective effect of H_2_S in rats with heart failure (HF). GYY4137 is a water-soluble H_2_S donor (102), which at physiological pH and temperature, releases low concentrations of H_2_S into in aqueous solution for several hours, conditions which simulate the time course of H_2_S release *in vivo* (103). In this way, GYY4137 protected the myocardium from I/R injury by reducing oxidative stress and apoptosis (104). DATS-MSN is a long-term sustained release H_2_S donor, which can prevent myocardial I/R injury (105). Compared with NaHS and GYY4137, Sun et al. (105) demonstrated that DATS-MSN provides superior cardiovascular protection, which may be related to the its capacity for long-term slow release of H_2_S. SPRC, which is a novel endogenous H_2_S water-soluble regulator synthesized by Zhu et al., promotes angiogenesis by activating signal transduction factors and transcriptional activators (106) (Figure 3).

### Antioxidant Effect of H_2_S

Studies have shown that ROS and Reactive nitrogen species (RNS), such as peroxides (·O_2_), can be eliminated by H_2_S at any time, and that the antioxidant activity of H_2_S is higher than that of cysteine, GSH and other antioxidants (107). However, the physiological concentration of H_2_S is much lower than that of other typical antioxidants (108). Nevertheless, exogenous H_2_S has been shown to have a strong antioxidant capacity and protect cells from damage in physiological systems exposed to ROS and RNS. In addition, despite the differences in concentration of gaseous signaling molecules, H_2_S is a small molecule with properties that allow it to pass freely through the cell membrane, thus enhancing its antioxidant activities compared with macromolecule antioxidants in the microenvironment (109). H_2_S exerts its antioxidant effects through extensive indirect signal conduction rather than direct effects on ROS/RNS. Thus, H_2_S treatment can provide far-reaching and long-lasting antioxidant protection in cells.

H_2_S exerts its antioxidant effects by regulating the expression and activity of classic antioxidants such as GSH and Thioredoxin (Trx). GSH, a tripeptide composed of glycine, glutamate, and cysteine, is one of the major antioxidants in cells. Furthermore, cysteine is synthesized from methionine via a thiogenic pathway to produce the key enzymes required for H_2_S synthesis (CSE and CBS) in each step of the catalytic reaction. Studies have shown that H_2_S promotes GSH production, thereby protecting the heart from oxidative stress (110). Trx is a small molecule (~12 kDa) containing a cysteine-glycine-proline-cysteine motif at its catalytic site. The two cysteine residues are the main sites for Trx oxidation and promote ROS reduction through thiol-disulfide exchange. Oxidized Trx is reduced by the Trx reductase and then reduced by NADPH. Trx activity has been shown to perform intracellular and extracellular functions in ROS elimination to protect against oxidative stress (111). A recent study showed that H_2_S treatment not only increased Trx expression in ischemic HF models, but also attenuated high-fat-induced left ventricular remodeling (112). Mice expressing dominant Trx-negative mutations did not respond to H_2_S treatment, indicating that the cardioprotective effect of H_2_S in this HF model is Trx-dependent. Finally, H_2_S has also been shown to regulate the expression of Trx interaction protein (TXNIP), which binds to and inhibits Trx activity, in endothelial cells (113).

As an important antioxidant stress transcription factor, Nrf2 regulates the expression of many antioxidant genes and cell protective genes. In a mouse model of myocardial ischemia, H_2_S preconditioning activated Nrf2 signaling, up-regulated the expression of antioxidant proteins HO-1 and thioredoxin 1, and reduced myocardial ischemic injury (99). It has been reported that myocardial mitochondria are the main sites of oxidative stress induction during myocardial ischemia-reperfusion (MI/R), with different responses to H_2_S regulation in suborganelles. Furthermore, MI/R damage to the submusculoskeletal mitochondria in rat heart plays an important role in reducing H_2_S mediated oxidative stress (114). The antioxidant function of H_2_S is due, in part, to the direct removal of ROS and/or inhibition of ROS production. Geng et al. (115) showed that H_2_S reduces lipid peroxidation by removing O_2_^**.−**^ and H_2_O_2_ in isoproterenol-induced damaged myocardium. In hypoxic/reoxygenation rat cardiomyocytes, H_2_S reduces ROS levels and protects the myocardium by inhibiting the activity of mitochondrial complex IV, enhancing the activity of superoxide dismutase (SOD) enzymes, such as Mn-SOD and CuZnSOD (116).

OS plays an important role in the pathogenesis of HF. Oxidative stress leads to apoptosis, which may cause damage to cardiomyocytes. H_2_S is an effective ROS scavenger and has a protective effect on HF. Sirtuin-1 (SIRT1) is a highly conservative nicotinamide adenine dinucleotide (NAD)-dependent histone acetyl enzyme that plays a key role in promoting cell survival under conditions of oxidative stress. Using NaHS as the H_2_S donor, Wu et al. (117), showed that NaHS increased SIRT1 expression under conditions of oxidative stress, and reduced H9C2 cardiomyocyte apoptosis via the SIRT1 pathway.

### Anti-apoptotic Effect and H_2_S

H_2_S has anti-apoptotic properties. *In vivo* studies have demonstrated that H_2_S activates pro-growth kinases, such as PKC/ERK1/2 and PI3K/Akt, and activation of PKC/STAT3 signals, leading to increased expression of anti-apoptotic molecules, such as Hsp90, Hsp70, and Bcl-2 (118, 119). Numerous studies (106, 120–125) have shown that H_2_S plays a protective role in ischemic myocardium through apoptosis-related signaling pathways. For example, Ning et al. (126) demonstrated that the H_2_S donor NaHS resisted AMI-induced apoptosis by upregulating the GSK-3β/β-catenin signaling pathway. NaHS reduced the apoptosis of myocardial cells after myocardial ischemia-reperfusion injury in rats by down-regulating the JNK signaling pathway (127). The CSE/H_2_S pathway mediates the protective effect of trimetazidine against hypoxia/reoxygenation induced apoptosis in H9C2 cells (128). Meng et al. found that pre-administration of GYY4137 as a H_2_S donor increased Bcl-2 expression in ischemic myocardium, while decreasing the expression of Bax, expression and caspase-3 activity, indicating that GYY4137 prevents myocardial ischemic injury (104) TUNEL assays showed that SPRC treatment for 30 min before hypoxia significantly reduced the apoptosis of isolated papillary muscle cells caused by hypoxia/reoxygenation injury and protected muscle morphology (129). Subsequent studies in HF rats showed that the expression of Bax induced by ischemia caused by ligation of the left coronary artery decreased the expression of Bcl-2, thereby triggering the activities of caspase 9 and caspase 3. The sustained release preparation of SPRC (CR-SPRC) increased Bcl-2 levels and reduced the levels of Bax, caspase 3 and caspase 9, thereby protecting myocardial cells (130). SPRC, which is also used as a H_2_S donor, enhanced cell activity, restored downstream gene expression regulated by GP130/STAT3, inhibited cell apoptosis, and antagonized mitochondrial dysfunction and intracellular Ca^2+^ overload in adriamycin-induced cardiac toxicity (130).

Moreover, genes involved in apoptosis regulation, such as microRNAs (miRNAs), can also act as important mediators that protect the ischemic myocardium. It was found that myocardial cell hypoxia/reoxygenation (HR) injury increased apoptosis, upregulated the expression of miRNA-1, and down-regulated the expression of Bcl-2 (131). However, H_2_S pretreatment reduced myocardial cells apoptosis after HR injury. Furthermore, this approach also down-regulated miRNA-1 expression and up-regulate Bcl-2 expression (131). MiRNA-133a is involved in the protective effect of H_2_S against myocardial cell apoptosis induced by ischemia/reperfusion (132). In addition, miRNA-208B-3P, miRNA-128-3P, and miRNA-320 are all related to myocardial ischemia and apoptosis, with down-regulation of miRNA-208B-3P inhibiting of myocardial injury caused by ischemia/reperfusion in rats (133), while inhibition of miRNA-128-3P protects myocardial cells from ischemia/reperfusion injury by upregulation of P70S6k1/P-P70S6k1 (134), and down-regulation of miRNA-320 inhibits apoptosis of myocardial cells and provides protection against myocardial ischemia and reperfusion injury by targeting IGF-1 (135). However, it is not known whether these miRNAs are related to H_2_S, and further research is needed to clarify this point.

### Anti-inflammatory Effects and H_2_S

The anti-inflammatory action of H_2_S results from the inhibition of leukocyte rolling, adhesion and flipping. In addition, it inhibits NF-κB and reduces the production of the inflammatory cytokines IL-1 and TNF-α (136). The levels of TNF-α, IL-1, and IL-6 in the serum of rats were shown to be increased after AMI, and the levels of ICAM-1 mRNA and NF-κB in the myocardial tissues were significantly increased. However, the levels of these factors decreased after NaHS, which inhibited the synthesis of inflammatory factors, such as IL-6 and nuclear transcription factors after MI in rats, thereby reducing myocardial injury and protecting myocardial tissue (137).

## Mechanism of H_2_ Protection Against Myocardial Ischemic Injury

### Induction of H_2_

H_2_ is widely distributed in nature. As a colorless and odorless reducing gas, it is not only small in size, but also freely crosses the blood-brain barrier, with no residue as a result of metabolism (138). Compared with NO and H_2_S, H_2_ has a smaller molecular weight and is more likely to enter the biofilm. H_2_ is less cytotoxic than other medical gases, and its low reactivity with other gases allows it to be mixed with other therapeutic gases, including inhaled anesthetics (139). Therefore, H_2_ is expected to become the fourth most important gaseous signaling molecule after NO and H_2_S (140). The synthetic sources of H_2_ are endogenous and exogenous. H_2_ is not normally produced in human cells due to the absence of enzymes with hydrogenase activity. However, under normal human physiological conditions, more than 12 L of H_2_ is produced daily, mainly by the fermentation of undigested carbohydrates produced by the microbiome (141). Studies have shown that, to play an antioxidant role, the content of endogenous hydrogen must be significantly higher than the minimum concentration of exogenous hydrogen (142). H_2_ is excreted in three main ways: through breathing, flatulence and metabolization by microorganisms in the colon (143). The human body can obtain exogenous H_2_ through inhalation and drinking or injection of hydrogen-rich water (144). Studies have shown that drinking hydrogen-rich water in daily life is beneficial for some chronic diseases (145). Nagatani et al. (146) found that intravenous hydrogen salt was safe and effective in 38 patients with acute ischemic stroke. In addition, eye drops or external products that produce H_2_ can be absorbed into the blood through the skin, thus representing a potential strategy for the use H_2_ to treat diseases (147). Intake of exogenous H_2_ has been shown to have a great effect on the body (1) in the studies of multiple organs or systems including the central nervous system (145, 148, 149), cardiovascular system (122, 150), lung (151, 152), renal system (145), liver (153), pancreas (154, 155), intestinal (156, 157). In the study of the cardiovascular system, H_2_ has been shown to H_2_ play an important role in myocardial ischemic injury (158).

### Antioxidant Effect of H_2_

Oxidative stress is the main cause of myocardial ischemic injury (159). Ohsawa et al. (158) found that H_2_ selectively reduced the levels of hydroxyl radicals and cytotoxic ROS to effectively protect cells, although H_2_ cannot reduce free-radicals by interacting with excessive ROS. This discovery provides a new strategy for the treatment of myocardial ischemic injury. In recent years, Nrf2 has been identified as a transcription factor closely related to the oxidative stress caused by H_2_. Xie et al. (160) evaluated the role of the Nrf2/HO-1 signaling pathway in ischemia induced in H9C2 myocardial cells *in vitro* through serum and glucose (SGD) deprivation. The results showed that SGD caused myocardial cell damage and down-regulated the Nrf2/HO-1 signaling pathway. In contrast, a H_2_-rich gas alleviated the cell damage caused by cell exposure to SGD and up-regulated Nrf2/HO-1. In addition, RNA interference mediated silencing of the Nrf2 gene, the influence of H_2_ on HO-1 induction and cardiac protection was significantly reduced.

In summary, H_2_ gas protects myocardial cells from myocardial damage caused by ischemia by eliminating ·OH free-radicals and activating the Nrf2/HO-1 signaling pathway. In addition, the Nrf2/ARE pathway also plays an important role in selective oxidation. Studies have shown that H2 can protect the myocardium by activating Nrf2-ARE signaling pathway (161, 162).

### Anti-apoptotic Effects of H_2_

During MI/R, oxygen free-radicals, calcium overload and MPTP opening lead to mitochondrial swelling and rupture, releasing apoptosis-inducing factors and apoptosis-related proteins, and further initiating the caspase cascade to induce programmed apoptosis (163). Recent studies have shown that the PI3K/AKT pathway is crucial for cardiomyocyte apoptosis (164). Chen et al. (165) found that high concentrations of H_2_ protected mouse hearts from ischemia-reperfusion injury by activating the PI3K/AKT1 pathway. Forkhead box protein O (FoxO) is downstream of PI3K/AKT and is inhibited by PI3K/AKT. Generally speaking, after FoxO is activated, the cells cycle is blocked in the G1/S phase and apoptosis is promoted. Therefore, hydrogen may regulate FoxO expression via the PI3K/AKT signaling pathway, thus playing an anti-apoptotic role (1). In addition, hydrogen-enriched saline also showed protective and antiapoptotic effects on MI/R injury by down-regulating the AKT/GSK3 signaling pathway (166) and upregulating the JAK/STAT signaling pathway (167).

### Anti-inflammatory Effects of H_2_

Zhang et al. (168) demonstrated that ischemia/reperfusion (I/R) induced elevated levels of the pro-inflammatory cytokines TNF-α and IL-1β in myocardial cells, which were attenuated by hydrogen-rich saline. Hydrogen-rich saline has an anti-inflammatory effect on the local MI/R injury in the heart. There are few reports on the protective effects of H_2_ against myocardial ischemia injury and inflammation, which requires further investigation.

## Study on the Mechanism of Myocardial Ischemic Injury by Combining no and H_2_S

Accumulating evidence indicates the potential coupling of NO and H_2_S at different levels. ZYZ-803, which is a novel synthetic H_2_S and NO co-donor, was developed by combining SPRC with furoxan. However, ZYZ-803 releases H_2_S and NO more slowly and in a more prolonged manner than SPRC and/or furoxan (169). ZYZ-803 has fewer side-effects and lower concentrations than SPRC and furoxan alone. The cardioprotective effect of ZYZ-803 was significantly stronger than that of the H_2_S and/or NO donors alone. Furthermore, ZYZ-803 releases H_2_S and NO by stimulating CSE and endothelial NO synthase (e-NOS), respectively, to produce physiological activity. Currently, there are few reports on ZYZ-803, most of which focus on the mechanism of angiogenesis (170) and vasodilation (171) and the protective effects against HF (172).

Chang et al. (120) used ZYZ-803 as the combined gas donor to study its anti-apoptotic effects on MI injury. By releasing H_2_S and NO, ZYZ-803 down-regulated the RIP3-CaMKII signaling pathway and alleviated ERS-related necrotic apoptosis after AMI. DL-propargylglycine (PAG) and e-NOS inhibitors of N(G)-nitro-L-arginine methyl ester (L-NAME), which inhibit of CSE and L-NAME, respectively, significantly inhibited the cardioprotective effect of ZYZ-803, while the inhibitory effect of PAG+L-NAME was more obvious. In addition, Wu et al. (171) found that blocking CSE and/or e-NOS inhibited the generation of H_2_S and NO produced by ZYZ-803 and reversed its cardiovascular protective effects. It has been reported that NO promotes CSE expression in vascular tissues and increases H_2_S levels, while L-NAME inhibits the vasodilatory effect of H_2_S (171, 173). Furthermore, H_2_S enhances the production of NO through calcium-dependent activation of e-NOS in endothelial cells (174). The generation of NO is obviously inhibited by CSE knockout, while CSE overexpression promotes the generation of NO (175). These data suggest that PAG not only inhibits CSE and reduces H_2_S, but also inhibits e-NOS activity and NO concentrations in HF. L-NAME not only inhibits e-NOS and reduces NO, but also reduces CSE expression and H_2_S levels. Thus, H_2_S and NO have synergistic effects, while H_2_S regulates the biological function of NO, and vice versa.

## Discussion

**Figure 4:**
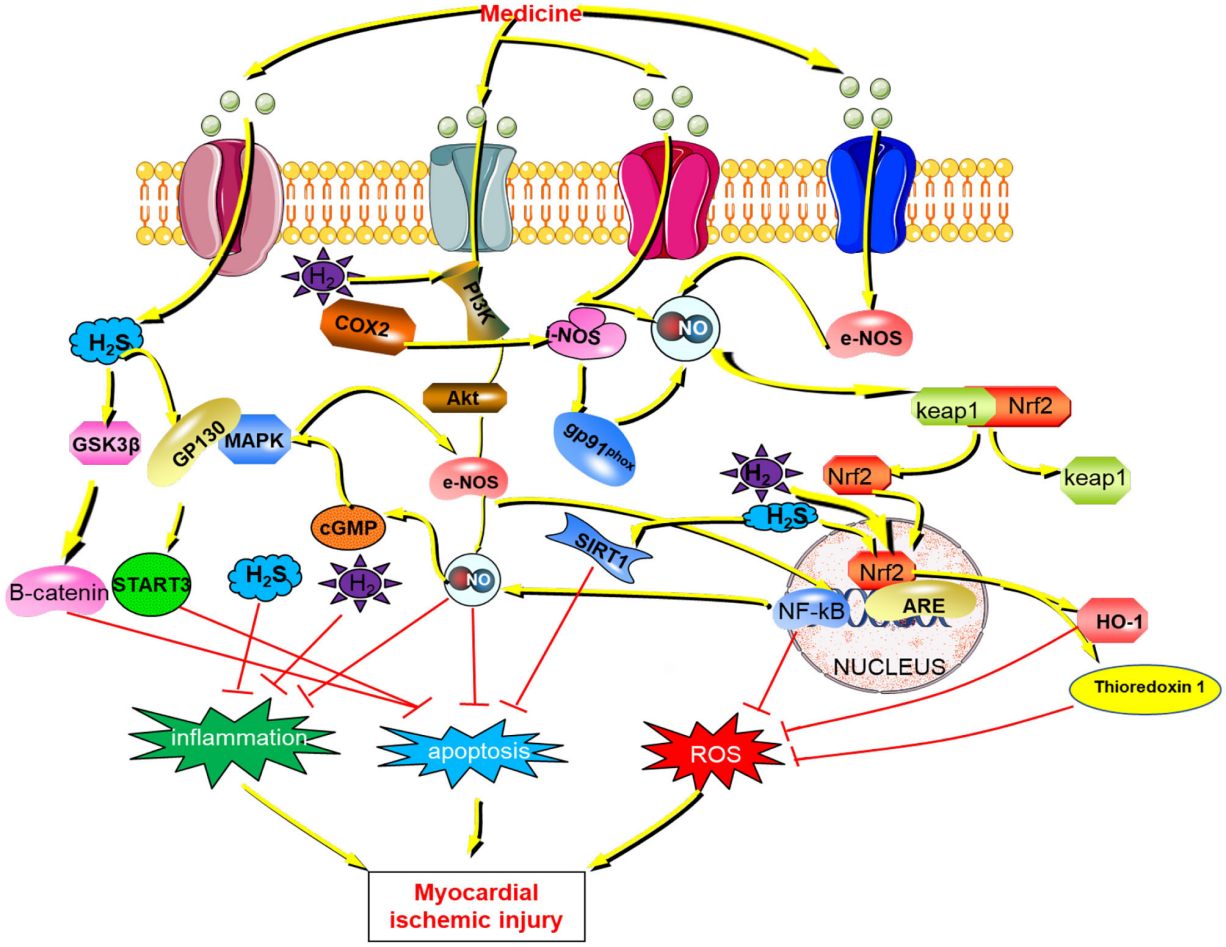
The mechanism of gaseous signaling molecules (NO, H_2_, H_2_S) against myocardial ischemic injury H_2_S, hydrogen sulfide; GSK3β, Glycogen synthase kinase-3β; GP130, Glycoprotein 130;STAT3, Signal transducers and activators of transcription 3; PI3K, class I phosphatidylinositol 3-kinase; COX2, Cyclooxygenase 2; AKT, Serine/threonine protein kinase; MAPK, mitogen-activated protein kinase; cGMP, cyclic guanosinc monophosphate; NO, nitric oxide; i-NOS, Inducible nitric oxide synthase; e-NOS, endothelial nitric oxide synthase; Keap1, Kelch Like ECH Associated Protein 1; Nrf2, Nuclear factor erythroid 2 p45-related factor 2; NF-kB, Nuclear factor-kappa B; HO-1, Hemeoxygenase-1; SIRT1, Sirtuin1; ROS, Reactive oxygen species.

In the past few years, substantial progress has been made in the field of gaseous signaling molecule donors. In this review, we have summarized the mechanisms by which gaseous signaling molecules (NO, H_2_S, H_2_) protect myocardial ischemia (Figure 4), including the gas co-donors that regulate gas molecules that protect against myocardial ischemic injury. Since most of the experimental articles listed in the review included normal and model groups, and the gaseous signaling molecule donor did almost no damage to normal cardiomyocytes, the off-target effect was rarely seen. However, when the concentration of gaseous signaling molecules such as H_2_S is too high, poisoning and other phenomena will occur. Therefore, targeting and other approaches can be adopted to increase the concentration of gas signaling molecules locally within the lesion to achieve the therapeutic effect of gaseous signaling molecules.

As a ubiquitous gas, NO also mediates and hemostasis and homeostasis, which enhances the protective effect on the heart. Studies have shown that vascular endothelial cells are the main cellular source of synthesized NO *in vivo*, and the synergistic effect of endothelial cells and platelet NOS is conducive to regulating platelet activation and inhibiting platelet adhesion and aggregation (176). NO also plays an important role in maintaining vascular tone and blood pressure homeostasis.

H_2_S inhibition of oxidative stress to protect cardiomyocytes is dependent on Nrf2-mediated induction of cellular protection genes. John et al. found that H_2_S induced upregulation of Nrf2 inactivates Keap1 through modification of C226 and C613 while Nrf2 controls CBS, CSE, and Sqrdl (sulfide: quinone reductase-like), which suggests the existence of a feedback loop between Nrf2 and H_2_S. H_2_S also protects the heart from oxidative stress through S-acidification.

Anti-inflammatory strategies are an important aspect of drug therapy for myocardial ischemic injury. On the one hand, inflammatory responses fundamentally affect the long-term and short-term performance of solid organ allografts; on the other hand, the transplantation process including the surgical trauma itself, in addition to the associated ischemia-reperfusion injury may lead to acute and chronic inflammatory responses that affect allograft function in the long-term (177). If the inflammation is resolved, the tissue can heal without sequelae. Otherwise, acute inflammation may become chronic, stimulating tissue remodeling and ultimately leading to fibrosis and loss of organ or tissue function (177). Therefore, drugs that effectively control inflammation and acomprehensive understanding of the mechanism of its action are urgently required. However, the pathways by which the gaseous signaling molecules (NO, H_2_S and H_2_) exert their anti-inflammatory effects in myocardial ischemic injury remain to be elucidated.

NO has long been recognized as a gaseous signaling molecule that acts independently; however, recent studies have shown that H_2_S is an important enhancer of NO in blood vessels. Specifically, H_2_S may act as a system enhancer for e-NOS/sGC/cGMP/PKG, mainly by stimulating NO release in stable and semi-stable pools, and by stimulating calcium mobilization to stimulate e-NOS activity. Similarly, when the biological effect of vascular NO system is weakened, the stimulatory effect of H_2_S on the e-NOS/sGC/cGMP/PKG system is weakened (178). These findings indicate that there is crosstalk between the gaseous signaling molecules, and that they do not function independently. Although there are few studies on the interaction between NO/H_2_ and H_2_S/H_2_ at present, advances in science and technology will facilitate relevant studies on these aspects.

To data, many donors of gaseous signaling molecules have been identified, although the exact concentration of gaseous signaling molecules in various samples is unknown, which hinders progress in this field of research. Therefore, an appropriate and accurate method is urgently required. In addition, gaseous signaling molecules such as H_2_S, specific inhibitors and stable donors are lacking. Due to the wide range of current inhibitor molecules and the instability of donor molecules, the actual changes in H_2_S concentrations conflict with the expected results.

Experimental studies have shown that gaseous signaling molecules with co-donor drugs, such as ZYZ-803, have a much better effect on the treatment of diseases than in individual drugs administered with gas signaling molecules. Therefore, gaseous co-donor drugs and their targets will be an important focus of research. Moreover, gaseous signaling molecules have shown certain therapeutic effects in many animal experiments related to myocardial ischemic injury. Clarification of the specific targets of gaseous signaling molecules in myocardial ischemic injury and evaluation in clinical applications is of great significance. It is believed that with advanced in scientific research technology and the continuous efforts of researchers, gaseous signaling drugs will soon enter clinical research.

## Summary

The aim of myocardial ischemic treatment is to relieve symptoms, reduce the incidence of angina pectoris and MI, and delay the development of coronary atherosclerosis. In recent years, myocardial ischemic injury has attracted increasing attention due to its high risk of death. The protective effect of gaseous signaling molecules on myocardial ischemic injury is self-evident, and clarification of the specific targets of gaseous signaling molecules in myocardial ischemia is of great and urgent significance for their clinical application. Hopefully, this review will serve as a reference for guidance of future research into the effects of gaseous signaling molecules on myocardial ischemic injury.

## Author Contributions

WW and TG wrote the manuscript. YM revised the manuscript. XC and YZ proved the manuscript. All authors contributed to the article and approved the submitted version.

## Conflict of Interest

The authors declare that the research was conducted in the absence of any commercial or financial relationships that could be construed as a potential conflict of interest.

## Abbreviations

AMI: Acute myocardial ischemia
ANG II: Angiotensin II
BH4: tetrahydrobiopterin
CABG: Coronary artery bypass graft
CAPE-oNO2: o-nitrophenyl ethyl caffeate
CBS: Cystathionine β-synthase
COX1: Cyclooxygenase-1
CSE: Cystathionine γ-lyase
e-NOS: Endothelial NO synthase
ER: Endoplasmic reticulum
FAD: Flavin adenine dinucleotide
FMN: Flavin mononucleotide
FoxO: Forkhead box protein O
GSH: Glutathione
GSRg3: Ginsenoside Rg3
GYY4137: morpholine-4-methoxyphenylmorpholine-morpholine-phosphodisulfate
HF: Heart failure
H2: Hydrogen
H2S: Hydrogen sulfide
HSP90: Heat shock protein 90
IL-1: Interleukin 1
IL-6: Interleukin 6
Keap1: Kelch-like ECH-associated protein 1
KMUP-1: 7-[2-[4-(2-chlorophenyl) piperazinyl] ethyl]−1
L-NAME: N(G)-nitro-L-arginine methyl ester
i-NOS: Inducible NO synthase
I/R: Ischemia-reperfusion
MI: Myocardial infarction
mPGEs1: Microsomal PGE synthase-1
3-MST: 3-mercaptopyruvate sulfurtransferase
NF-κB: Nuclear transcription factor-kappa B
n-NOS: Neuronal NO synthase
NO: Nitric Oxide
Nrf2: Nuclear factor erythroid 2 p45-related factor 2
OS: Oxidative stress
PAG: DL-propargylglycine
PCI: Percutaneous coronary intervention
PG: Prostaglandin
PI3K: Phosphoinositide 3-kinase
QSQP: Qishen yiqi drop pill
RNS: Reactive nitrogen species
ROS: Reactive oxygen species
SAC: S-ally-l-cysteine
SGD: Serum and glucose
SOD: Superoxide dismutase
SPRC: S-propargyl-cystine
SR: Sarcoplasmic reticulum
TNF-α: Tumor necrosis factor-α
Trx: Thioredoxin
TUNEL: Terminal deoxynucleotidyl transferase (TdT) dUTP nick-end labeling
TXNIP: Trx interaction protein
XD: Xanthine dehydrogenase
XO: Xanthine oxidase.
